# The evolution of marine dwelling in Diptera

**DOI:** 10.1002/ece3.7935

**Published:** 2021-07-24

**Authors:** Nina Pak, Stephanie Wu, Joel F. Gibson

**Affiliations:** ^1^ Department of Environmental Science, Policy, & Management University of California Berkeley California USA; ^2^ Entomology Collection Royal BC Museum Victoria BC Canada

**Keywords:** ancestral state reconstruction, coastal Flies, macroevolution, PASTML, WoRMS

## Abstract

Marine dwelling in Diptera has been relatively unexplored and the frequency of transitions to the marine environment and the evolutionary history remain poorly understood. By reviewing records from the World Register of Marine Species and using ancestral state reconstruction methods, we build on the fly tree of life phylogeny and ecological descriptions of marine life history. Our ancestral state reconstruction analyses suggest marine dwelling is lacking as an ancestral trait for the most recent common ancestor to Diptera. While many transitions in Empidoidea, Sciomyzoidea, Tipulomorpha, and Culicomorpha seem to have been gradual, other transitions in Tephritoidea and Tabanomorpha were found likely to have been stochastic occurrences. From the collection of 532 marine species, we reveal several independent transitions to the marine environment throughout the fly tree of life. Considering the results from our analysis, we outline potential adaptations for marine flies and discuss the barriers of colonizing the marine environment and the implications to the mechanisms for salt tolerance.

## Graphical abstract

Highlights of the marine fly tree of life show several independent transitions to the marine environment. The distribution of marine‐dwelling Diptera has challenged the notion that marine flies are a rare phenomenon. Multiple mechanisms for adapting to the marine conditions are likely at play.

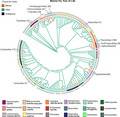

## 1 INTRODUCTION

Habitat transitions among marine, freshwater, and terrestrial environments give insights into the challenging barriers in colonizing new habitats. While major habitat transitions have been relatively rare, the ability to cope and interact with environmental changes across ecosystems provides an opportunity to examine the processes of colonization and evolutionary diversification. For example, transitioning from marine to freshwater habitats has allowed multiple radiation and speciation events for many taxa (Lee & Bell, 1999; Waters et al., 2020). On macroevolutionary time scales, this transition from marine to freshwater has occurred more frequently than the transition from freshwater to land (Grosholz, 2002). In addition, nonmarine animal clades, when compared to marine clades, have shown higher rates of diversification (Wiens, 2015). Particularly, arthropods have been successful in exploiting habitats, radiating into the greatest species abundance of any extant phylum (Lee, 2016; Thomas et al., 2020). Insects were among the first animals to colonize and exploit terrestrial and freshwater ecosystems (Misof et al., 2014; Vermeij, 2020). The increasing number of insect phylogenies, both within and between orders, and the extensive work relating to major drivers of diversification brings opportunities to examine the broad‐scale patterns of ecological transitions.

The species of Diptera are an ecologically diverse group and have colonized the aquatic environment (Adler & Courtney, 2019). Proposed phylogenies have illustrated the evolution of an aquatic life history in many families (Wiegmann et al., 2011). Within studies of aquatic dipterans, those focusing on marine flies are sparse in comparison with the breadth of freshwater dipteran literature. The marine paradox highlights this gap in the literature: despite the estimated 1.6 million kilometers of coastline around the world, which can range from intertidal zones, estuaries, salt marshes, and dunes to rocky and sandy beaches, marine‐dwelling insects are rare—estimated to be about 2,037 species of insects in the sea and less than 1,000 fly species associated with the marine environment (Ayyam et al., 2019; Merritt et al., 2019; Vermeij, 2020). These numbers contrast with an estimated 5.5 million species of insects in total globally (Stork, 2018). As many of these coastal habitats are at risk and in decline across the world due to environmental changes and human activity, a greater understanding of the dipterans living in the marine environment, such as the coast and intertidal zones, would help to interpret responses to changes in sea levels and salinity as a result of global climate change (Beaumont et al., 2014; Kefford et al., 2016). An opportunity to examine the marine environment is also a chance to understand saline environments and the common means of coping with salt and stress.

Marine dwelling is a complex trait for many reasons. Salinity has been a source of stress for many animal phyla, shaping distributions and influencing community structures of ecosystems (Arribas et al., 2014). Evolving salt tolerance may mitigate the effects of osmotic and ionic stress of salinity while also allowing populations to escape predation, reduce competition, and avoid water loss (Arribas et al., 2014). However, Diptera are not well known as osmotically sensitive organisms. Previous literature documenting marine adaptation in Diptera is nonexistent in contrast to other insect orders, with the exception of Cheng’s (1976) compilation of articles on marine arthropods (Cheng, 1976). In contrast, relatives of Diptera have developed several pathways to combat salt. For example, species of Crustacea and Coleoptera have been used in several studies examining osmoregulatory systems in both the freshwater and marine environments (Lee, 2016; Pallarés et al., 2017).

In this paper, we focus on marine‐dwelling Diptera. Almost all marine‐dwelling insects live in the intertidal zone, spending a fraction of their lives underwater, most commonly in the egg, larval, and pupal stages. We can expect salt‐tolerant or halobiont (organisms that develop only in saline habitats) species to occur across the fly tree of life as rare events (Szadziewski, 1983). No species of Diptera is known to spend their entire life cycle fully submerged in the sea.

The purpose of this research is to outline an evolutionary model for understanding the few marine‐dwelling fly species currently known. We do not make the assumption that salinity plays the only role in determining whether an organism can live in a marine environment, but the implications of inhabiting these environments can illuminate mechanisms and adaptations to saline environments. Macroevolutionary investigations and ancestral state reconstructions can reveal mechanisms that allow for evolutionary transitions (Edwards & Donoghue, 2013). By examining the phylogenetic distribution of marine‐dwelling species of flies, we explore what the macroevolutionary perspective can reveal about the evolution of marine dwelling and to the extent of salt tolerance.

Here, we take advantage of the fly tree of life proposed by Wiegmann et al. (2011) to address how local‐scale ecology (e.g., inhabitating a marine or terrestrial environment) relates to the diversity of Diptera, and to consider the phylogenetic distribution of marine‐dwelling Diptera (Wiegmann et al., 2011). A macroevolutionary approach takes a broader view, where the focus is not on particular species or environments but on detecting any general patterns in the evolution of a trait by comparing a large number of lineages using phylogenetic analyses. We can estimate how often marine dwelling has evolved and its evolutionary history by examining the distribution of marine flies on a phylogeny.

## 2 METHODS

### 2.1 WoRMS annotation

We compiled a list of 145 Diptera families based on the family‐level fly tree of life with 206 species (Wiegmann et al., 2011). To find published reports of Diptera that have at least one life‐history stage in a marine environment, we relied on the World Register of Marine Species (WoRMS, www.marinespecies.org). In WoRMS, species are attributed to the following environments: marine, brackish, freshwater, terrestrial, and combinations thereof (Costello et al., 2015; Horton et al., 2021). The mission of WoRMS, aside from being a global scale biodiversity inventory, is to integrate global marine species information and standardize the species names recorded worldwide since 2008 (Costello et al., 2015; Ng et al., 2017). Taxonomic information is checked by WoRMS’s taxonomic experts as a quality assurance process (Ng et al., 2017).

We coded each fly family for ecological traits: marine dwelling and aquatic life history. Fly families containing at least one species from the WoRMS database were coded as “Marine” (Yes = 1) and those without as “Other” (No = 0). Families with an aquatic life history from the literature were recorded as well (Yes = 1/No = 0) (Adler & Courtney, 2019; Wiegmann et al., 2011). Marine families with 4 species or fewer were designated as ambiguous for potentially being misclassified or observed as rare events (See Table S2). The dataset of all records is available as electronic supplementary material in file DataS1. The database of WoRMS records for Diptera was collected in December 2020.

### 2.2 Ancestral state reconstruction

We investigated the evolution of marine dwelling in Diptera to assess how frequently the trait was gained or lost across the proposed phylogeny (Wiegmann et al., 2011). Using a rooted phylogeny as a scaffold and the presence or absence of at least one marine species within the fly family as a character, we employed PastML for ancestral state reconstruction (ASR) for discrete characters (Ishikawa et al., 2019).

Based on the marginal posterior probabilities approximation (MPPA) in PastML, we performed ASR by maximum likelihood. The analyses were performed based on the F‐81 model, the Jukes Cantor model, and the estimate from tips (EFT) model. In an EFT model, equilibrium frequencies are calculated based on the tip state proportions. The Jukes Cantor model uses equilibrium frequencies where all frequencies between states are equal, instead of being estimated. Alternatively, the F‐81 model allows marginal posteriors to be inferred with an optimized scaling factor. We then assessed the optimal model selected by the Akaike information criterion (AIC) based on Table S1, resulting in the EFT model (Akaike, 1974). Our assessment of the marine diversity, based on number of species from WoRMS records, was then combined with ASR analyses.

## 3 RESULTS AND DISCUSSION

### 3.1 Taxonomic distribution: Is marine dwelling confined to a few key specialist clades or does it occur throughout the dipteran phylogeny?

**FIGURE 1:**
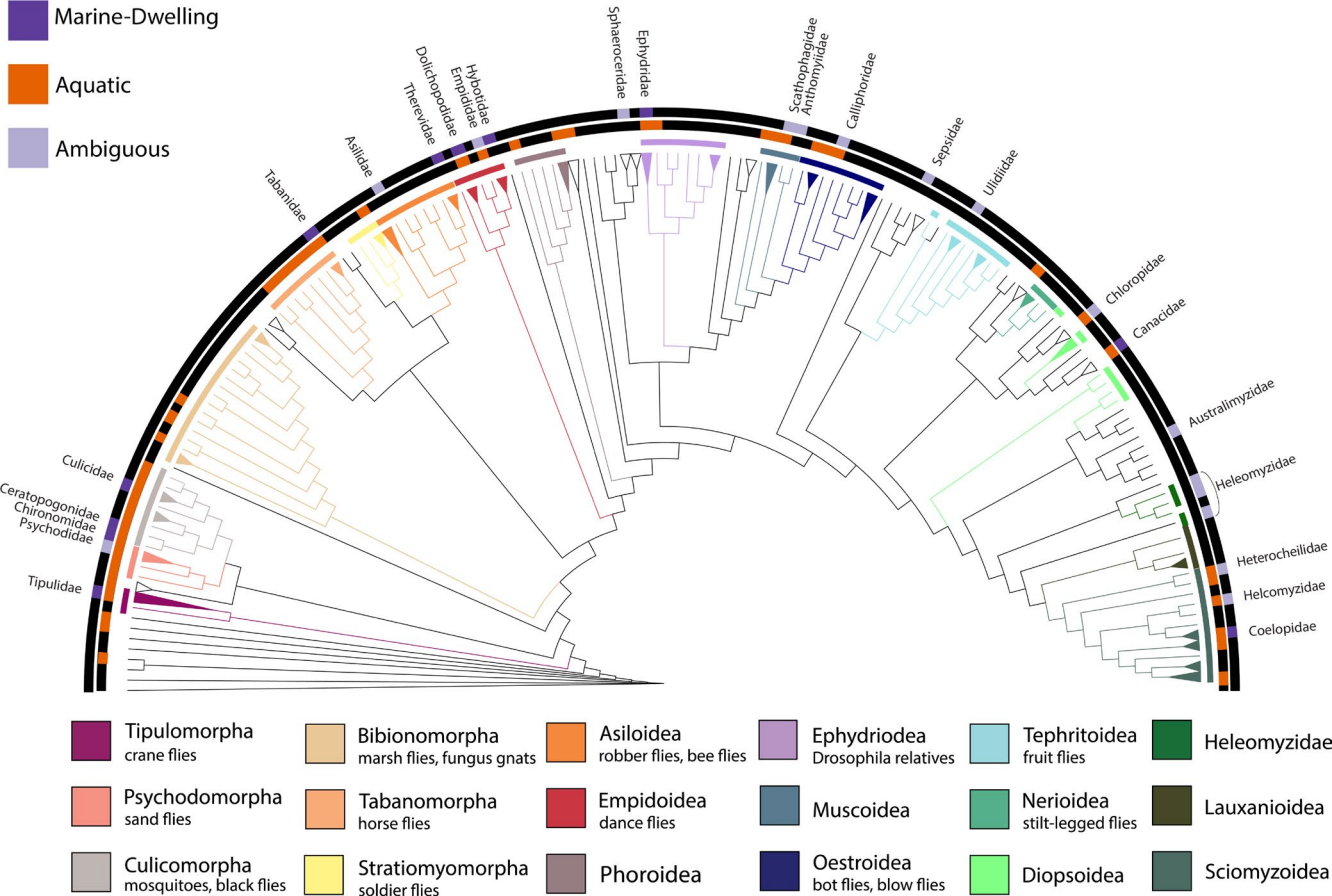
Diptera phylogeny from Ref. (Wiegmann et al., 2011) with marine dwelling, aquatic life history, and ambiguous (families with less than 4 marine species) ecologies labeled. Infraorders and superfamilies are colored

Ecological classifications can be examined in Data S1, which list the number of fly families that have been known to occupy the aquatic environment based on WoRMS. Table S2 includes the number of Marine species in each family. From our full dataset, we present results of combined traits of marine dwelling, aquatic life history, and ambiguous ecologies in Figure 1. WoRMS and the tree from Wiegmann et al. (2011) do not include all flies in existence and all environments they inhabit, but both use the most complete set of fly genetic and ecological data ever collected thus far. By definition, aquatic Diptera includes species with at least one life history stage in either a marine, freshwater, or a combination of both in these environments. Specifically, it is a strong association with bodies of water that encompasses aquatic dipterans, not necessarily saline conditions (Merritt et al., 2019). In addition, for defining marine insects, Gibson and Choong (2021) state, “A marine insect species is a species that spends at least one of its developmental stages habitually in a marine habitat, that must feed, either as larvae or adults, on other marine organisms, or that has an ethology that is intimately linked to marine organisms, such as a reliance on other insects that depend on marine organisms as hosts”.

We used the WoRMS database to quantify which families had the highest proportions of marine Diptera. We investigated whether marine Diptera are distributed randomly across the fly tree of life with over 200 Dipteran taxa. Marine dwelling had multiple independent origins. Twenty‐five families (17% of fly families, 25/145) have at least one species living in the marine environment based on the WoRMS database. The estimation could be far greater when including fly groups that live outside of the sea but in salty environments, such as saline lakes and other inland saline environments. While marine origins are more likely in some clades than in others (like Empidoidea and Sciomyzoidea), marine‐dwelling flies are dispersed widely on the phylogeny. The groups Bibionomorpha, Stratiomyomorpha, Phoroidea, and Nerioidea all lacked any marine species, while other groups had one or a few lineages. Of the 532 WoRMS’ designated marine‐dwelling Diptera, the family Therevidae had 180 records, which was the highest number of records. Overall, only three families, Therevidae, Ephydridae, and Hybotidae, had more than 50 species. Nonetheless, fourteen of the twenty‐five families were designated as ambiguous, suggesting that marine‐dwelling flies are the minority in many Dipteran families, generally constituting a small percentage of the described species (see Table S2).

After examining the distribution of marine and aquatic designations across the fly tree of life, a few patterns emerge. Not all aquatic fly families include marine‐dwelling species and not all marine‐dwelling species are in families most notable for being aquatic (Figure 1). Only 36% (9/25) of marine flies were within otherwise nonaquatic groups (Figure 1). These families are Asilidae, Therevidae, Hybotidae, Sphaeroceridae, Anthomyiidae, Sepsidae, Ulidiidae, Australimyzidae, and Heleomyzidae (Figure 1).

### 3.2 Ancestral state reconstruction: When did marine dwelling evolve in diptera?

**FIGURE 2:**
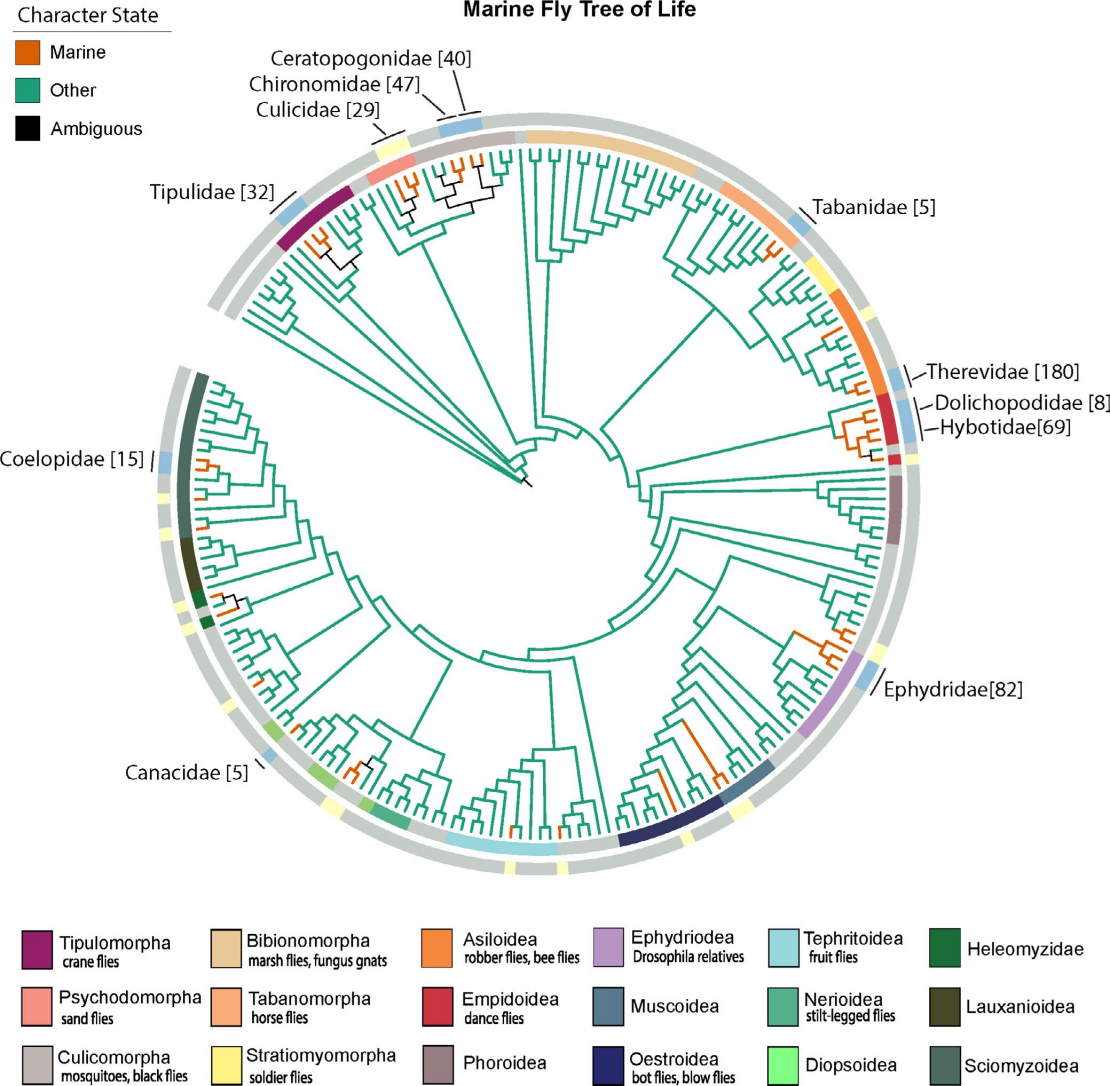
Ancestral state reconstruction for marine‐dwelling flies under EFT model using the Diptera phylogeny from Wiegmann et al. (2011). Colored labels show infraorders and several superfamilies. Character states were made based on posterior probability estimates. The marine families based on WoRMS records were categorized into groups with 5 or more species (blue) and those with 4 or fewer species (yellow). Bracket numbers show number of species. Complete list of species, ecological descriptions, and families are found in Data S1. Figures were made through PASTML (Ishikawa et al., 2019; Letunic & Bork, 2019)

For habitat transitions between marine and other environments, we used maximum likelihood ancestral state reconstruction method in PASTML (Ishikawa et al., 2019). Under the three models of evolutions (JC, F‐81, and EFT), the estimated ancestral state constructions were largely congruent, but EFT was concluded to have the best AIC score. AIC scores can be found in Table S1. We estimate that marine dwelling has evolved at least 20 times in the family‐level phylogeny of Diptera (Figure 2). Our analysis is at the family level, although shifting to marine habitat has been gained within a superfamily several times (e.g., in the Empidoidea and Sciomyzoidea).

Our ancestral state reconstruction demonstrated that marine dwelling is likely to have evolved independently multiple times across the Diptera phylogeny. The most recent common ancestor of Diptera was inferred to have lived in an aquatic environment, but not necessarily a marine environment. In the EFT model, the earliest transition could have occurred in the Tipulomorpha, particularly within the Limoniidae lineage with a posterior probability (PP) value of 0.69. Families within Tipulomorpha are notable for their aquatic life history in freshwater ecosystems, and thus, transitions to the marine environment could have taken place via the freshwater environment instead of the terrestrial environment. Other lineages in Nematocera, specifically within Culicomorpha and Psychodomorpha, exhibited marine dwelling in families like Psychodidae (PP 0.58), Culicidae (PP 0.80), and shared between Ceratopogonidae and Chironomidae (PP 0.54). A direct transition to the marine environment could have taken place among many nematoceran lineages, but had several accelerated reversions to freshwater and terrestrial habitats. This could explain why the state of the most recent common ancestor of lineages in Culicomorpha and Psychodomorpha was ambiguous, but estimated with a posterior probability of 0.37 for marine dwelling.

Among the Empidoidea, fly families Dolichopodidae (PP 0.95) and Hybotidae (PP 0.96) shared a common ancestor, likely to have been capable of living in a marine environment (PP 0.80). Shifts to the marine environment could have originated from a freshwater environment for the notably aquatic family, Dolichopodidae, while in Hybotidae, the transition to the marine environment may have been derived from the terrestrial environment, as there are several terrestrial sister groups (McAlpine et al., 1981). This suggests differences in barriers to colonize the marine environment, and that multiple mechanisms for adapting to the marine conditions are likely at play.

In addition, in the more recent diverging groups in Sciomyzoidea, especially the families Coelopidae, Helcomyzidae, and Heterocheilidae, all had marine species within the WoRMS database records. These families share notable aquatic ecologies, with the implication of a progression to the marine environment directly from the freshwater environment. With these lineages, losing the ability to live in the marine environment may not have occurred throughout evolutionary history. Given the absence of evidence for terrestrial species within these families, we earmark them and Canacidae as Diptera families where the most recent common ancestor specialized in the marine environment. These four families may represent the best case for “fully marine adapted” Diptera lineages.

The majority of transitions to the marine environment in Empidoidea, Tipulomorpha, Culicomorpha, and Sciomyzoidea seem to have been gradual. Other transitions in the Tephritoidea (fruit flies) and Tabanomorpha were found to have likely been stochastic occurrences within the lineage. The ancestral state reconstruction suggests that shifts to the marine environment have occurred within more recently diverging Dipteran clades; marine dwelling is lacking as an ancestral trait but arose several times.

### 3.3 The marine fly tree of life

Our distribution of marine‐dwelling Diptera has challenged the notion that marine flies are a rare phenomenon. The diversity of marine‐dwelling Diptera has not been demonstrated before in the context of the fly tree of life. Marine dipterans appear across the fly tree of life, lacking prominence across any one specific infraorder or superfamily. The lack of determination of an ancestral marine origin for any one clade could be explained by the limited number of species within the phylogeny or by the lack of literature exploring marine life histories and the ecophysiological adaptations to the aquatic environment. Future work will no doubt reveal the ecological mechanisms that have allowed transitions to the marine environment as well as address the literature gaps in the knowledge of their aquatic ecologies and life histories.

While the currently known marine Diptera species are taxonomically widespread, some groups of marine flies lack any overlap with known aquatic groups. Groups, labeled as ambiguous with 4 or fewer species (Figure 2), are suspected to include transitions to the marine environment as a random occurrence. We highlight the families containing more than 4 species, suggesting these groups include species where the marine environment may play a significant role in its diversification (See Table S2). In addition, we note that several of the designated marine fly families differ in their overall species‐level diversity and proportion of marine species (See Table S2). For instance, Canacidae (beach flies) is estimated to have 307 global species, where over 90% inhabit the coast and the intertidal zone (Munari & Mathis, 2010). In smaller families like Coelopidae (commonly known as kelp flies with nearly 30 species) and Helcomyzidae (12 species), all described species are exclusively marine, feeding on red and brown algae on the coast. In comparison, Therevidae have over 1,100 species within the family, but the marine species make up a minority of the known global diversity and its larval habitats more commonly range from arid desert environments to open woodlands. Despite what geographical or environmental factors may have influenced these differences in current diversity, future work must consider marine dwelling beyond the family level.

Insect species included in the WoRMS database range in their degree of ecological, physiological, and anecdotal data. WoRMS contains an ongoing list of marine dipterans, but relies entirely upon published literature and the scientific community to self‐report ecological descriptions of species. Some groups with well‐known marine life history such as *Thoracochaeta* (Sphaeroceridae: Limosiinae; (Marshall, 1982; Hodge et al., 2017)), *Oedoparena* (Dryomyzidae; (Gibson & Choong, 2021)), *Telmatogeton* (Chironomidae: Telmatogetoninae; (Brodin & Andersson, 2009; Lorenz Simões et al., 2020; Nondula et al., 2004)), *Thalassomya* (Chironomidae: Telmatogetoninae; (Qi et al., 2019)), and *Tanytarsus* (Qi et al., 2019) were missing from the overall list of marine species in WoRMS. Similarly, genera like *Fucellia* (Anthomyiidae: Anthomyiinae; (Kaczorowska, 2005)) and *Pontomyia* (Chironomidae: Chironominae; (Huang & Cheng, 2011)) were given marine designations in WoRMS as a family but were not among the taxa examined in the fly tree of life (Wiegmann et al., 2011). For example, Chironomidae is represented in the phylogeny by a single taxon although the family includes several thousand species, including many known marine dwellers (Morley & Ring, 1972). As more taxa become part of phylogenies, ecological descriptions and life history observations will become essential in further understanding the diversification of these marine lineages.

Our analysis suggests that the distribution of marine dwelling across fly families can be due to multiple evolutionary patterns and individual adaptations in different lineages. This cannot replace detailed understanding of marine adaptations at the physiological level or examination of the particular strategies employed by different species, but the hope is that the macroevolutionary patterns could reveal some of the underlying evolutionary forces that shape biodiversity and distribution of marine Diptera, which might then prompt more fine‐scale studies that examine the links in more detail.

### 3.4 Potential macroevolutionary mechanisms

Examining the phylogenetic distribution of marine‐dwelling flies is a stepping stone to understanding the evolution of salt tolerance in Diptera. Salt tolerance may be necessary before transitioning to the marine environment or other saline inland habitats and may also be difficult to persist as a trait across macroevolutionary scales. One reason is that an investment in salt tolerance could be costly. The production of compatible solutes or transport of ions could be a costly evolutionary tradeoff—using up resources that could be put into other functions or behaviors like growth and reproduction. It is important to consider that transitions from one environment to another do not signify that organisms become independent of the environment they have partially left (Bromham, 2015; Vermeij, 2020).

Based on our ancestral state reconstruction, we observe marine dwelling can be a labile trait with an enhanced rate of loss—it is often gained, but then is typically lost several times. This scenario is likely given that marine dwelling may be a result of a combination of several ecophysiological traits and no one particular trait confers salt tolerance or stress tolerance within the marine environment. Within some families, particularly Canacidae and Coelopidae, adapting to the marine environment may have been gained early on in the evolutionary history and then occasionally lost as some of these lineages transitioned to freshwater environments (O’Grady & Pak, 2016). The absence of evidence of reversions to the terrestrial environment within these families suggests that their most recent common ancestor specialized in marine environments. Future work will investigate whether colonizations of the marine environment were via the freshwater or the terrestrial environment and whether marine dwelling within a family is difficult to evolve or modify following Dollo's law (Marshall et al., 1994; Wagner et al., 2018).

**FIGURE 3:**
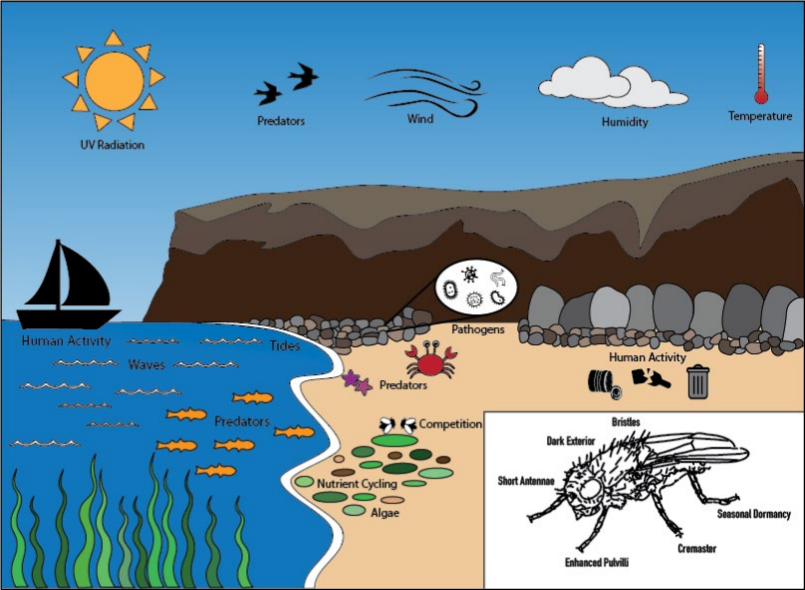
Stressors (abiotic and biotic) and physiological adaptations for marine flies are shown. Figure was adapted from Ref. (Brodin & Andersson, 2009)

Marine dwelling can vary across lineages based on the degree of stress—acute or chronic. Some putative associations with salt tolerance include cuticle formation, osmoregulation, fat storage, desiccation tolerance, drought tolerance, ion transportation, sodium and chloride pumps, detoxification, and speciation. Marine dwelling as a trait could be explained by several dependencies on other stress‐tolerant traits. Potential physiological adaptations to a wide range of marine environmental challenges include developing dark shades on the cuticle, short antennae, cremaster (hook structures), enhanced pulvilli (tarsal pads for clinging to surfaces), additional bristles, seasonal dormancy, and other changes in phenology (Brodin & Andersson, 2009; Vaz et al., 2021). Stress from the environment, as abiotic factors, could be in the form of UV radiation, wind, variable temperatures, fluctuating tides, humidity, and water surface tension from the nearby sea (Dionisio‐Sese et al., 2001; Ikawa et al., 2012; Peace, 2020). Biotic factors that may be shaping marine fly populations include predation by birds, fish, and other beach fauna, competition from other marine organisms, the degree of kelp, algae, and other forms of vegetation, as well as the pathogens and endosymbionts inhabiting the beach (Rechsteiner et al., 2018; Wickham et al., 2020). In addition, human activity has influenced the state of both terrestrial and marine environments, possibly disrupting natural processes of nutrient cycling and vegetation growth (Nielsen et al., 2004). We summarize these environmental factors in Figure 3.

We speculate different physiological and environmental constraints for flies inhabiting the saline/hypersaline inland habitats (e.g., Ephydridae in Great Salt Lake). It seems likely that other factors may be equally or more critical in the saline inland habitats due to the absence of competition from marine arthropods (i.e., crustaceans). Future work will investigate the potential differences between colonizations in the marine environments and the inland saline habitats, and extend to examining the relationships between diversification rates and degrees of salinity.

## 4 CONCLUSIONS

Understanding the abilities and constraints of dipteran populations to adapt to salt and the marine environment will become more critical as humans continue to impact the world's aquatic resources through climate change, landscape modification, and pollution, resulting in increasingly stressful habitats for aquatic Diptera. Through a macroevolutionary approach, we are making the case that marine Diptera may not be as rare as generally assumed, but that it also is very unlikely to be an adaptation that arose only once within Diptera. Through classifying habitats and studies, we conclude that the abilities of these species to locally adapt to coastal habitats require more work on less observed species and several evolutionary adaptions may be involved.

Our understanding of the evolutionary processes leading to this adaptation is also in its infancy. We summarize the existing knowledge on this subject and present a possible framework toward the development of an evolutionary model of dipteran adaptation to the marine environment and, by extension, salt. While no published list of marine flies will be complete, due to poor knowledge of salt tolerance in certain families and geographical regions, this will be the most extensive database of known marine flies.

## CONFLICT OF INTEREST

The authors declare that there is no conflict of interest.

## AUTHOR CONTRIBUTIONS

**Nina Pak:** Conceptualization (equal); Data curation (equal); Formal analysis (equal); Funding acquisition (equal); Investigation (equal); Methodology (equal); Project administration (equal); Resources (equal); Software (equal); Supervision (equal); Validation (equal); Visualization (equal); Writing‐original draft (equal); Writing‐review & editing (equal). **Stephanie Wu:** Investigation (equal); Resources (equal); Validation (equal); Visualization (equal); Writing‐review & editing (equal). **Joel F. Gibson:** Data curation (equal); Formal analysis (equal); Investigation (equal); Resources (equal); Supervision (equal); Validation (equal); Visualization (equal); Writing‐review & editing (equal).

## Supporting information

Data S1

Supplementary Material

Appendix

## Data Availability

Annotations, WoRMS records, and Fly Families (Data S1 file) can be found on Dryad (https://doi.org/10.6078/D1799Z).
