# Supplementary material for: The evolution of marine dwelling in Diptera
Source: Ecol Evol. 2021 Jul 24;11(16):11440–8. doi: 10.1002/ece3.7935 (PMC8366842; doi:10.1002/ece3.7935)
Supplement: Supplementary file 3 — Appendix [file ECE3-11-11440-s001.docx]

**Appendix**

Supplementary Table 1: AIC scores for Evolution Models for Ancestral State reconstruction

|  | EFT | JC Model | F81 |
| --- | --- | --- | --- |
| Likelihood | -96.43208237632474 | -94.83937580867357 | -94.64274869909319 |


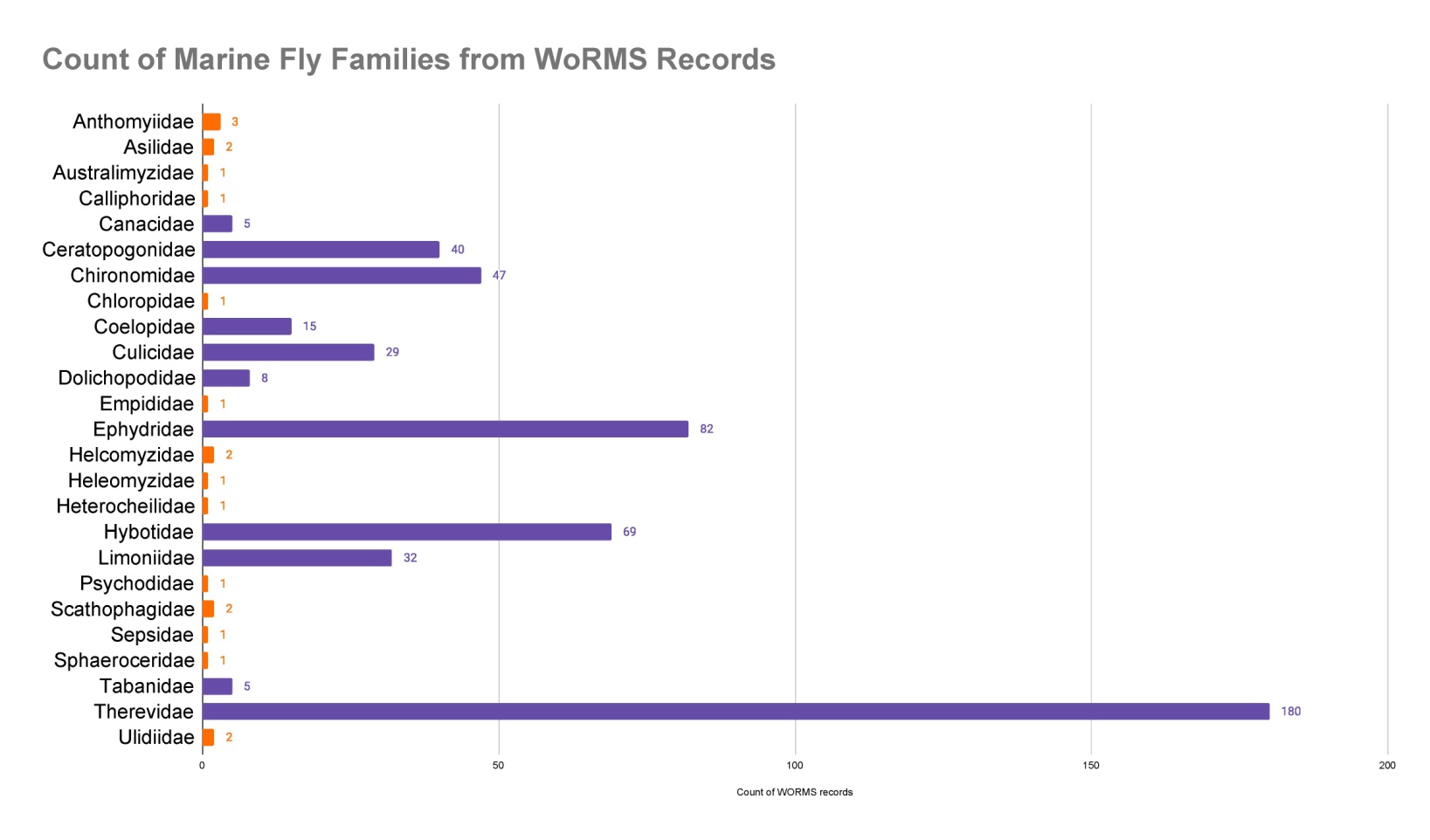


Supplementary Table 2: List of WoRMS families and the number of records within a fly family. Orange labels show families with 4 marine species or fewer. Purple indicates families with more than 5 species. For these families with more than 5 species of marine species, we consider them most notable for their marine dwelling species.

Data S1: List of Fly Families (annotations of marine and aquatic life histories) can be found on Dryad (<https://doi.org/10.6078/D1799Z>).
